# Designing stem cell niches for differentiation and self-renewal

**DOI:** 10.1098/rsif.2018.0388

**Published:** 2018-08-29

**Authors:** Hannah Donnelly, Manuel Salmeron-Sanchez, Matthew J. Dalby

**Affiliations:** The Centre for the Cellular Microenvironment, University of Glasgow, Glasgow G12 8QQ, UK

**Keywords:** mesenchymal stem cell, stem cell niche, self-renewal, differentiation, biomaterials, regenerative medicine

## Abstract

Mesenchymal stem cells, characterized by their ability to differentiate into skeletal tissues and self-renew, hold great promise for both regenerative medicine and novel therapeutic discovery. However, their regenerative capacity is retained only when in contact with their specialized microenvironment, termed the *stem cell niche*. Niches provide structural and functional cues that are both biochemical and biophysical, stem cells integrate this complex array of signals with intrinsic regulatory networks to meet physiological demands. Although, some of these regulatory mechanisms remain poorly understood or difficult to harness with traditional culture systems. Biomaterial strategies are being developed that aim to recapitulate stem cell niches, by engineering microenvironments with physiological-like niche properties that aim to elucidate stem cell-regulatory mechanisms, and to harness their regenerative capacity *in vitro*. In the future, engineered niches will prove important tools for both regenerative medicine and therapeutic discoveries.

## 1. Introduction

Mesenchymal stem cells (MSCs) have the ability to both self-renew and differentiate, yielding daughter cells that are essential for tissue maintenance and repair. Unlike embryonic stem cells (ESCs), MSCs must tightly balance specialization in response to regenerative demand and retention of a stem cell pool throughout life and this balance is controlled by the niche environment e.g. the bone marrow [1]. Further, perhaps because of their pericyte/perivascular origin [2], MSCs have the ability to suppress the immune response and reduce inflammation. These properties make MSCs ideal therapeutic candidates. Potential clinical applications are wide ranging, from underpinning tissue regeneration for the treatment of trauma (tissue engineering/regenerative medicine [3,4]) to novel cancer therapies (homing to tumours [5] and then delivering drug/gene therapies [6,7]) and new transplant protocols (providing immune-suppressed environments allowing tissue engraftment [8–10]).

However, since their discovery in 1974 [11] there have been less clinical success stories than first imagined for MSCs, currently there are only a small number of other adult stem cell (ASC) therapies approved in the clinic. Perhaps of greatest success, are bone marrow transplants which for over 50 years have been harbouring haematopoietic stem cells (HSCs) to treat leukaemia and lymphoma patients, and more recently autoimmune diseases such as multiple sclerosis [12]. In 2015, the use of limbal stem cells from the eye were approved in Europe to repair cornea injury and restore sight [13].

MSCs are easily harvested from autologous bone marrow, fat or umbilical cord and have shown promise *in vitro* and *in vivo* with their ability to form bone and cartilage as well as through their immunomodulatory capacity; indeed, this capacity is finding use in e.g. islet transplant procedures [14]. Autologous MSC-based products, e.g. PREOB and Bonofill are in advanced clinical trials. Further, companies are starting to look to market allogenic MSC-based products, such as Trinity ELITE/Evolution, AlloStem, Osteocel Plus, Cellentra VCBM.

MSCs mediate the fine balance between differentiation, proliferation, self-renewal and quiescence through cell-intrinsic regulatory networks (box 1). However, only in concert with their specialized microenvironment do they retain their unique properties. This local microenvironment is termed the *stem cell niche*. First described four decades ago by Schofield [16], it refers to the extrinsic physical and functional factors that feedback to mediate cell behaviour. ASC niches have been identified in multiple human tissues, including HSCs in the bone marrow [17], neural stem cells in the subgranular and subventricular zones [18], epidermal stem cells in the hair follicle [19,20] and corneal limbus [21] and intestinal stem cells (ICSs) in the base of the epithelial crypt [22]. The perivascular origin of MSC is likely the factor that means that MSCs are found in different anatomical locations e.g. bone marrow, adipose tissue and close to the corneal limbus [2].

Box 1.

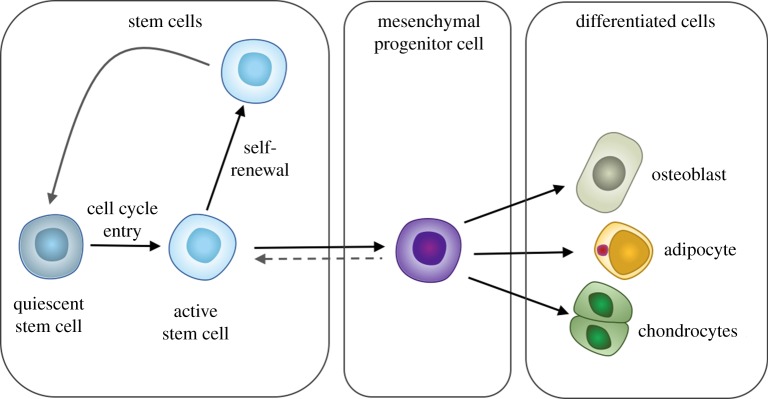
**Box 1.** Model of the organization of stem cells and tissue-specific mesenchymal progenitors. Multipotent stem cells exist in homeostasis between quiescence and an activated state. Activation, and entry to the cell cycle, occurs upon tissue damage or other physiological stimuli. Upon activation (regenerative demand), multipotent progenitor cells with transit amplification capacity arise. These progenitors are the precursors to the tissue-specific mature cells, for example osteoblasts, adipocytes and chondrocytes in the mesenchymal stem cell compartment. The activated stem cell and its daughter cells differentiate or can return to a quiescent state once the tissue repair or other physiological process is complete. This homeostasis between quiescence and self-renewal is tightly regulated to avoid transformations and to retain a viable stem cell pool throughout the life of the organism [15]. (Online version in colour.)

Model organisms have served as excellent tools for investigating stem cell niches, however they do not convey the complexity of the mammalian niches that have proven difficult to access and visualize, leaving many niches incompletely defined [23]. Mechanistic studies on model systems, such as *Drosophila melanogaster* and *Caenorhabditis elegans*, allow combinatorial approaches of molecular, genetic, systems and cell biology methods that have contributed greatly to understanding stem cell behaviours. *Drosophila* offer simple experimentation on well-characterized structures such as the fly gonad, which contains a niche environment with germ-line stem cells that are actively dividing. Owing to genetic and cell-biological methods that are uniquely available to fly biologists these simple systems render powerful tools. How similar fly and mammalian niches are would ultimately require parallel understanding of structures and functions, however some similarities can be drawn, for example, from conserved signalling pathways and cell types that will ultimately prove key for underpinning mechanisms in the mammalian niche [23,24].

Many challenges remain about what niche components are fundamental for retaining stem cell properties—how and what is being controlled, and for what purpose? Aims to address these challenges rely on advances in technologies that will allow the recapitulation of the niche outside of the body. Such technologies will offer greater insight into components, and cell-intrinsic and extrinsic interactions that regulate stem cells in specific microenvironments. This will allow us to understand what questions we need to answer to exploit these cells using biotechnological expansion approaches for therapeutic potential. As biomaterial technologies advance, answers to these questions are being elucidated, with the ability to construct and manipulate ‘de novo’ niches and harness the differentiation potential of stem cells.

**Figure 1.**
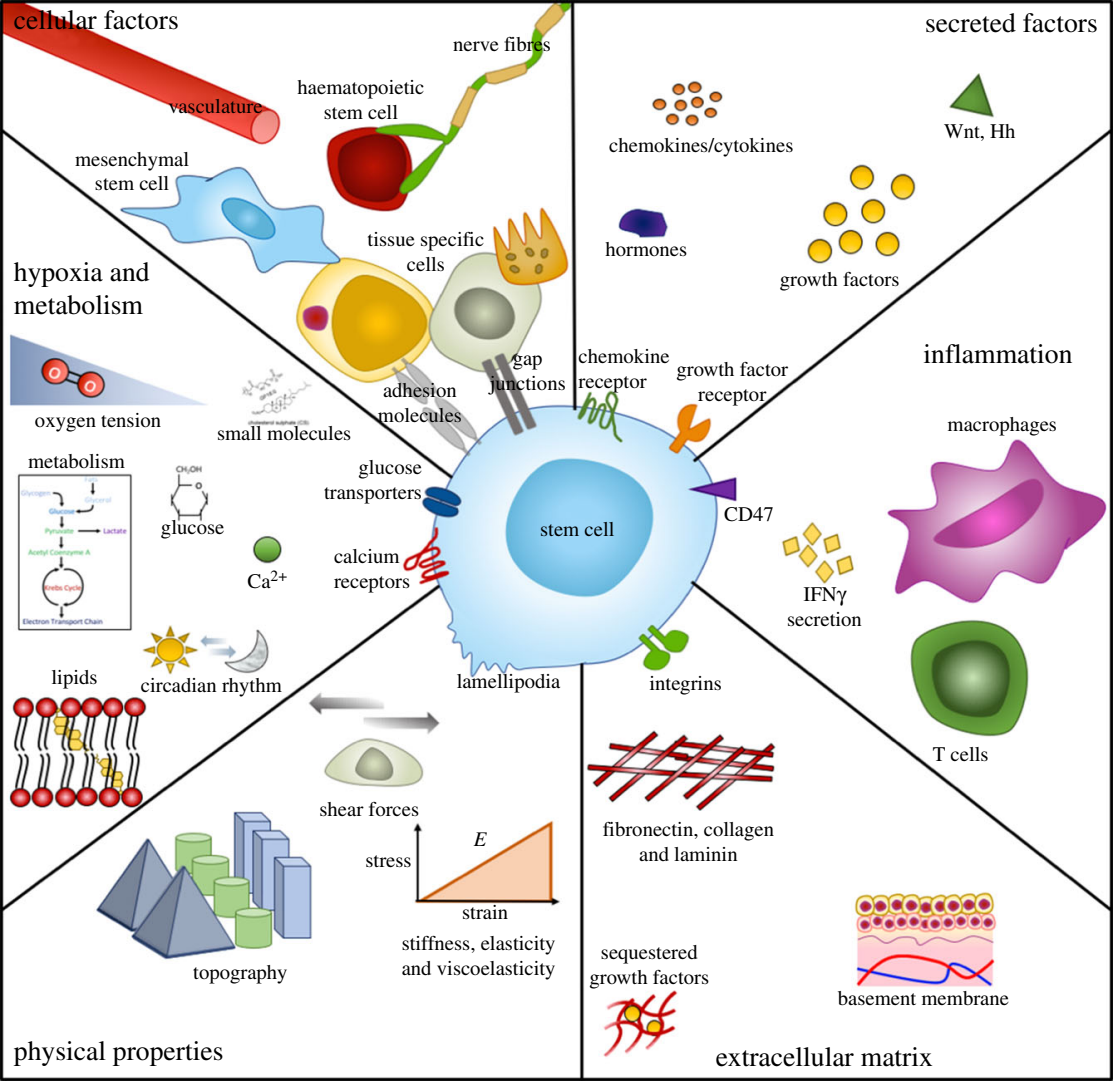
Parameters of the stem cells and their niches. Niches are multi-factorial and complex microenvironments that are unique and specific to function, however many principle parameters of niches are shared. Generally, they are comprised of physical and dynamic factors such as heterologous cellular components and cell–cell interactions, soluble and secreted or membrane bound factors, immunological activation and response, extracellular matrix (ECM) protein components and structures, physical architectural parameters, oxygen tension and metabolic control. Adapted from reference [25]. (Online version in colour.)

Biomaterial (surfaces, tissue engineering scaffolds), biofabrication (microfluidics, three-dimensional bioprinting) and bioreactor (physiological environment) techniques hold the potential to allow us to construct, deconstruct and investigate the important components of cellular microenvironments. Such approaches could evolve the development of both reductionist stem cell interfaces allowing high throughput analysis and discovery and, perhaps more importantly, non-animal technologies (NATs) that recreate tissue complexity and reduce costly/inefficient animal experimentation. The challenges, however, are great. The niche, as highlighted in figure 1, is a complex environment. It is notable that in small molecule drug discovery, the drive for high throughput, overly simplified cell models that do not recreate cell niches and animal testing in non-human models have fuelled the productivity crisis where large numbers of drug candidates are being taken forwards, many to ultimately fail in clinical trial. Only 43% of fails are not predicted by traditional *in vitro* and pre-clinical *in vivo* screens and move into clinical trials [26]. This is driving Pharma to look to NATs [27], built using human cells and likely requiring the tissue complexity that stem cells can produce. Such systems that can be used to predict drug mechanism, toxicity and efficacy require understanding of cell (stem cell) niche environments and techniques borrowed from regenerative medicine to direct the cells.

Here we review recent progress in the area and give a forward look on the development of artificial niches, with particular focus on MSCs. First, we discuss how biomaterial technologies have developed our understanding of cell–substrate interactions, and consider important factors in a cells' niche that allow us to differentiate stem cell populations for potential use in regenerative medicine. We then discuss how this understanding has led to recent advances in harnessing the capacity of stem cell self-renewal for prospective use in stem cell transplants and for immunosuppression. Finally, we provide an outlook on how combinations of such techniques provide opportunities for the generation of complex artificial stem cell niches.

## 2. Differentiation

Stem cell niches maintain self-renewal/quiescence [28]. However, this dynamic and multicellular environment must also signal for differentiation as part of regenerative processes. Cues in these environments are complex. Mechanical [29], physical [30], chemical [31], spatial [32] and temporal [33] cues ranging across many magnitudes—for example from subcellular level forces from the extracellular matrix (ECM), to organismal level in response to gravity [34]. These properties all inherently have both cell intrinsic and cell extrinsic effects, resulting in extensive effects on stem cell function.

### 2.1. Topography

To understand how properties of the biomaterial interface, such as stiffness, topography and chemistry can regulate cell behaviour, we must first consider how cells adhere to substrates. The architecture of a cell's microenvironment contains stimuli ranging from the micro to the nanoscale; microscale features are in the range of the size of the cell itself and result in whole-cell responses such as alignment of cells with topographical features, known as contact guidance [35]. However, nanoscale features present a multitude of cues that are several orders of magnitude below that of the cell [32].

Cell adherence to substrates is typically through integrins, transmembrane receptors that tether to the ECM, which itself forms an intermediate layer of proteins adsorbed on the surface of materials exposed to serum [36,37]. Integrins are heterodimeric proteins (containing α- and β-subunits) that ligate to peptide motifs on ECM proteins, for example the arginine, glycine and aspartic acid (RGD) tripeptide [38]. These interactions cause intracellular signalling cascades, typically G protein activation leading to phosphorylation of myosin light chain kinase (MLCK) through Rho-associated protein kinase (ROCK), increasing actin–myosin contractility causing integrin clustering and cell adhesion formation [39,40]. Adhesion formation is dynamic, cells use unbundled, actin-driven membrane projections, filopodia, to probe the external environment. It has been shown that filopodia can follow contact guidance cues down to 10 nm in height [41]. At the sub-10 nm height scale nanoscale projections have been detected, evidencing the great sensitivity of cellular sensing. It is noteworthy that at this sub-10 nm scale, contact guidance was not observed, just feature interactions [42].

Control over adhesion size has been achieved using lithography strategies to create nanopatterned substrates with controlled size, shape, spacing and symmetry in a variety of materials; patterns have included nanopits [43–45], nanopillars [46] and nanogrooves [47]. Control of these nano-features allows control over adhesion size, number and spacing. It has been shown that large, super-mature, adhesions (greater than 5 µm long) are required for osteogenesis of MSCs [48]. By creating substrates that promote increased adhesion size, intracellular tension is also increased and this conformational change is linked to mechanical changes in the cytoskeleton, which can transfer tensile (contractile) forces to the nucleus, perhaps via cytoskeletal tensegrity [49,50], and increased intracellular tension is linked to osteogenesis [48]. Such changes in nucleus shape can consequently affect chromosomal arrangements [51–53], and these changes can potentially impact stem cell phenotype [54].

Such alterations in cell adhesion, cytoskeletal organization and mechanotransductive cell fate changes are likely to be driven by the topography–protein interface. Indeed, if fibronectin (FN), a major cell-adhesive protein of the ECM is absorbed onto nanopit patterned surfaces, FN adsorbs within the pits and it was seen that cells probed these pits with filopodia, leading to ‘nanoimprinting’ of the pits on the cell membrane, an effect that was not observed when the substrate was not coated in FN [55]. Nanoimprinting has been shown to be cell-adhesion mediated, with adhesion to topographical features leading to mimics of the topography in the basal cell cytoskeleton [56]. If the integrins are blocked then nanoimprinting cannot occur [56], indirectly demonstrating the importance of the ECM on cell response to shape. This suggests that the topography-driven changes in cell cytoskeleton organization and adhesion are mediated by protein adhesive interface and cells interact with this interface dependent on the topography [55].

Cell adhesion and subsequent spreading that governs size and shape influence physiological processes such as cell survival [57]. Using microcontact printed ECM islands of decreasing size, it has been shown that cell confinement governs control over growth and death, with small areas that restrict spreading leading to apoptosis [57]. Since then, this technique has been employed to confine MSCs in specific morphologies, controlling adhesion and intracellular tension [39,40]. On ECM islands/shapes where the MSCs remained rounded, they were unable to form mature adhesions, leading to adipogenic lineage commitment. By contrast, ECM islands/shapes and sizes that allowed spreading, promoted actin–myosin contractility and mature adhesion formation drove MSCs to undergo osteogenesis [39,40]. The actomyosin tension of the cytoskeleton contributes to this geometric control, which is biophysically linked through adhesion formation governed at the nanoscale by changes in plasma membrane. It is thus further notable that modulation of the plasma membrane lipid assembly can regulate intracellular signalling and thus stem cell fate [58].

Topographical RGD coupled substrates have been used to decouple adhesion requirements for cell spreading. As integrins ligate, they are coupled to the actin cytoskeleton. Through activation of G-proteins inducing actin–myosin contraction, the integrins are gathered together leading to mature adhesion formation comprising many integrins. Using nanocolloid particles with one RGD motif covalently linked to each colloid, it has been demonstrated that when RGD is at a density of less than 70 nm apart, integrin gathering can occur; above this density integrins cannot gather together and form mature adhesions [59,60]. Using electron beam lithography approaches to create groups (dimers to heptamers) of RGD within 70 nm of each other, it was found that the small clusters separated beyond gathering distance, whereas tetramers of gathered integrins were required for complete cell spreading—i.e. functional adhesions [61].

**Figure 2.**
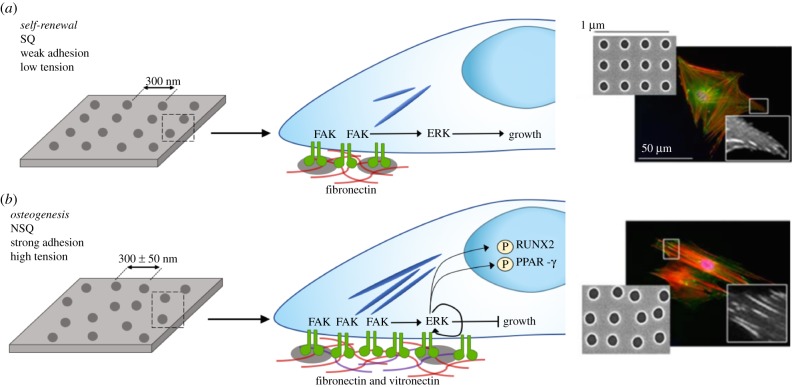
Topography to control MSC adhesion for self-renewal and osteogenesis. (*a*) Self-renewing MSCs adhere more-weakly than osteo-committed cells, leading to lower levels of integrin-mediated signalling through focal adhesion kinase (FAK), retaining levels of extracellular signal-regulated kinase (ERK1/2) to support growth but not differentiation. (*b*) MSCs undergoing osteogenesis require larger adhesions, increased FAK activation elevates ERK1/2 activation to levels required for lineage commitment, increasing intracellular tension, activating Runt-related transcription factor 2 (RUNX2), a key regulator of osteogenesis, while simultaneously inactivating adipogenic regulator peroxisome proliferator-activated receptor gamma (PPAR-y). Fluorescent images show a marked increase in adhesion size on near square (NSQ) compared with square (SQ) surfaces. Adapted from reference [43]. Copyright © 2018 American Chemical Society. (Online version in colour.)

**Figure 3.**
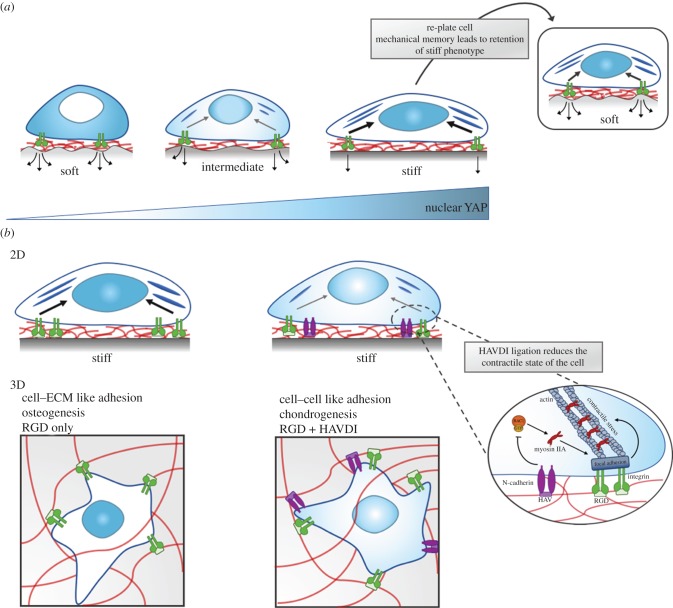
MSC mechanosensing of ECM stiffness cues. (*a*) Cell mechanosensing through integrin–ECM (in this case fibronectin RGD domain) detect changes in underlying matrix stiffness, as matrix stiffness increases so does the cytoplasmic-to-nuclear import of Yes-associated protein and its transcriptional co-activator (YAP/TAZ). YAP/TAZ is a master transcriptional regulator of many functional downstream effects, including differentiation of MSCs. MSCs will preferentially differentiate into osteoblasts on stiff matrices, and soft tissue types (such as adipose tissue) on soft matrices, hence increased nuclear import of YAP/TAZ on stiff substrates attenuates osteogenic differentiation of MSCs. In MSCs that are preconditioned on a stiff substrate, and then re-plated to a softer substrate, YAP/TAZ signalling remains active, and the cells remain committed to an osteogenic differentiation profile, i.e. they retain mechanical memory. (*b*) Modification of materials to contain specific peptide sequences can modulate mechanosensitive pathways. By addition of N-cadherin-based interactions, that mimic cell–cell contact (HAV motif—histidine, alanine and valine), pathways triggered by soft substrates are activated, and the contractile state of the cell is reduced. Whereas integrin-based interactions mimic cell–ECM contact (RGD—arginine, glycine and aspartic acid), leading to focal adhesion formation, which generates intracellular tension. HAV/N-cadherin interactions inhibit Rac-GTP levels, decreasing myosin IIA–actin interactions, reducing the recruitment of proteins to integrins to form focal adhesions, and hence reducing the contractile force generation on the cytoskeleton. Cells thereby behave as though on a softer substrate if HAV/N-cadherin ligation is present. In three-dimensional materials, peptide modifications such as this can mediate control over osteogenic or chondrogenic differentiation. Chondrocytes typically reside in pairs, meaning cell–cell interactions are preferable to cell–matrix interactions, i.e. presence of cadherin binding sites leads to chondrogenesis. Adapted from references [65,66]. (Online version in colour.)

This topographical control over how cells adhere to substrates has been used to control either self-renewal or osteogenic differentiation of MSCs, using topographies that can be remarkably similar. For example an electron beam lithographically defined pattern that permits out-of-niche self-renewal is comprised of pits with a diameter of 120 nm, depth of 100 nm and centre–centre spacing of 300 nm in a square lattice (SQ) [44]. Adding just ±50 nm offset from the centre position, changing the surface to near-square (NSQ) changes MSC fate to osteogenesis [45]. As noted above, adhesion size is quite different on each surface, with MSCs forming smaller adhesions and less intracellular tension on SQ compared to NSQ [43] (figure 2). It is interesting to note that cells on the NSQ surface change their endogenous ECM output from FN to vitronectin [62]. Vitronectin has been associated with increasing cells' ability to bridge gaps in the ECM, this means that if enough integrins are gathered in two close locations, intracellular linker proteins, such as vinculin, can span the gap in-between the two locations, even if there are no integrin ligands present in the gap; this is called bridging and vitronectin is a more effective bridging protein than FN [63]. We further note that the ECM is a complex mixture of proteins and that interaction with other receptors can also elicit changes. For example, the HAVDI sequence (histidine, alanine, valine, aspartic acid, isoleucine) can interact with cadherin receptors, key factors in cell–cell adhesion, and cause loss of intracellular tension, even in the presence of abundant RGD, through blocking the g-protein RAC [64,65] (figure 3*b*).

Osteogenic nanotopographies were originally developed with orthopaedic implants in mind, where patterns alone would guide cell fate. Such implants are typically made from titanium and its alloys. For example, hip replacements are stabilized by stems into the bone marrow of the femoral canals. The bone marrow, once disrupted, can no longer function effectively as a niche and the stem cells differentiate in response to the implant, but mainly into soft tissue forming fibroblasts. Fibroblasts are the default differentiation of the MSC, with MSCs originally identified as fibroblastic colony forming units [67]. This soft tissue encapsulation leads to micromotion and ultimately implant failure. It is thus notable that topographical features that drive osteogenesis, such as disordered (but not random) nanoscale patterns developed using electron beam lithography in polymer substrates have gone on to be featured in titania (the oxide of titanium) using polymer demixing to provide disordered masks for anodization processes [68–70]. It is further notable that such topographies retain osteogenic properties as they translate into metals [71,72]. However, better implant integration is only one problem with orthopaedic implants—infection is perhaps a greater problem and can lead to catastrophic implant failure. It is thus notable that bactericidal nanotopographies have started to be developed using high-aspect ratio nanofeatures [73]. Such nanofeatures can be tuned to support osteogenesis as well as kill bacteria [74], especially if the topographical approach is twinned with chemistries that can facilitate enhanced eukaryotic cell adhesion in order to also promote bone formation [75].

### 2.2. Mechanics

The ECM and surrounding cell junctions have a major physical influence in transmitting forces between cells, which ultimately regulate intracellular signalling pathways and therefore fate [29,66,76,77]. Cells can intrinsically generate mechanical forces within their environment, for example actin–myosin contractility leading to matrix remodelling [78,79]. Equally, mechanical force can come from extrinsic sources, such as tensile, compressive forces or shear stresses [29]. Whether individually or collectively, these mechanical forces impact and regulate cellular behaviour.

Intrinsic and extrinsic mechanical forces guide early embryo development, even from as early as the blastocyst stage, when remodelling of cell–cell junctions is driven by intrinsic cell forces to relieve tension as the embryo transitions through germ-band elongation. This remodelling of junctions is not determined by external forces at tissue boundaries but rather depends on myosin II-dependent spatial reorganization leading local forces at cell boundaries [80]. Later in development, the mechanical properties of the ECM regulate organ morphogenesis and development, where cell layers are organized into defined structures by traction forces on the ECM providing the template for organ growth [29]. This remodelling and development continues into adulthood as tissues maintain structure and function. One recent study highlighted how epidermal stem cells regulate this process through biomechanical signalling, where local crowding from dividing stem cells deforms cell shape and stress distribution, triggering differentiation of the neighbouring cell [77]. Owing to the complexity of this communication between cells and the environmental mechanical milieu, biomaterial strategies have played a key role in elucidating how these cues affect stem cell behaviour. By deconstructing complex environments and taking early reductionist approaches we are beginning to understand how mechanical force regulates cell behaviour.

Much research on MSCs, has previously been carried out on tissue culture plastic, or on other stiff and planar substrates. However, many stem cell niches often have low stiffness and are three-dimensional. Hydrogel systems are optimal for investigating mechanobiology due to their unique properties. Natural or synthetic polymers can be physically or chemically cross-linked in a controlled manner to produce hydrogel systems with tuned degradability, hydrophilicity and stiffness. The water that fills the space between the macromolecules leads to a degree of flexibility similar to that of natural tissues, making them both biocompatible and biomimicking [81]. Thus, hydrogels provide optimal systems for understanding cell response on or in soft substrates that are more physiological-like. Original work from Engler *et al.* used polyacrylamide (PAM) gels of tuneable matrix stiffness to guide stem cell fate through distinct tissue lineages; neural at 1 kPa, muscle at 12 kPa and bone at 30 kPa. This simple tuneable system has somewhat set the pace over the last decade for the unravelling and exploiting the biological mechanisms linked to mechanoregulation of MSCs [82]. Recent studies show stiffness also plays a role in cell migration [83], proliferation [84] and spreading [85].

Physiological interfaces *in vivo* exhibit gradients of stiffness, such as those at tissue junctions or at pathological boundaries, e.g. neuromuscular junctions and the tumour boundary [86]. Isolated cells are known to migrate to regions of different stiffness, ‘durotaxis’, the axis of migration depends upon the cell type; with stem cells known to migrate to regions of increasing stiffness [87,88]. Cancer cell lines, meanwhile, have been shown to have a variable relationship with substrate stiffness [89,90]. Multicellular clusters exhibit durotaxis, even if isolated cells do not. Groups of epithelial cells were found to migrate towards stiffer regions—cells atop stiffer substrate are able to gain better traction than on softer regions through integrin binding, intercellular junctions and the action of myosin motors are then able to contract neighbouring cells resulting in collective movement to firmer ground [91]. These observations highlight the need to think about both the inherent substrate stiffness and also the underlying stiffness gradient. Recently, a method of polymerization control has been developed to allow the user control over a stiffness gradient of PAM gels, using unreacted cross-linker and monomer in a prepolymerized hydrogel sink resulting in a tunable matrix ranging from 0.5 to 8.2 kPa mm^−1^. This allows for an *in vitro* model spanning the *in vivo* physiological and pathological range that can facilitate investigation of a range of mechanical signals on one surface [86]. Other studies have combined two biomaterial strategies to achieve tissue interface-like differentiation of MSCs. By engineering a hydrogel at a stiffness in the boundary of cartilage, but lower than that of bone, and combining this in a modular system with an osteogenic topographically patterned substrate, this approach enabled anisotropic differentiation of MSCs from a single source down chondrogenic and osteogenic lineages, similar to the interface of articular cartilage and bone found at the end of long bones [92].

Elucidating the mechanism behind what micro- and nanoscale properties cells sense at the cell–material interface of substrates of given stiffnesses has been a matter of some debate. Differentiation of MSCs on polydimethylsiloxane (PDMS) and PAM hydrogels of varying stiffness (0.1 kPa–2.3 MPa) with a covalently attached collagen coating were used to show that cell spreading and differentiation were unaffected by differing PDMS stiffness, with MSCs committing to osteogenic lineages. PAM substrates of high elastic modulus also exhibited osteogenic differentiation of MSCs, whereas this potential was lost on softer PAM substrates, decreased ERK/MAPK signalling was observed, suggesting MSCs were unable to form stable focal adhesions at this low modulus. Trappmann and colleagues used differences in ECM tethering to explain this effect, pore size on PAM substrates, but not PDMS, decreased almost twofold on soft substrates, proposing that varying pore size alters collagen tethering and thus local stiffness i.e. lower collagen anchoring density enhanced cell spreading and therefore differentiation [93].

On the other hand, work from the Engler group systematically modulated porosity of PAM substrates, without altering stiffness, to control ligand–substrate tethering. MSCs were cultured on these substrates and stiffness was indeed found to be the driving factor for cellular spreading and differentiation. Varying the degree of ECM–protein anchoring reaffirmed this indicating bulk stiffness of two-dimensional matrices as the main driver of cell response, irrespective of protein tethering and porosity [94].

Despite the exact rules for mechanical control remaining to be fully resolved, elucidation of cell signalling is emerging. Osteogenesis of MSCs on stiff substrates is regulated by the nuclear translocation of Yes-associated protein and its transcriptional co-activator (YAP and TAZ), which consequently activates the osteogenic transcription factor runt-related transcription factor 2 (RUNX2) [95]. Preserved nuclear compartmentalization of YAP/TAZ is observed when MSCs are cultured on stiff substrates for long culture periods (approx. 10 days), meaning they lose the ability to respond to softer matrices and remain committed to an osteogenic differentiation profile, suggesting MSCs possess a mechanical memory [95] (figure 3*a*). YAP/TAZ activation, until recently, was thought to be exclusive to the Hippo signalling pathway [96], however using PAM gels of various stiffness a recent study highlighted its direct mechanical activation [50]. By varying substrate stiffness it was found that if forces from adhesion and/or the cytoskeleton are high enough (above 5 kPa) stress fibres reinforce the cytoskeleton, in turn, mechanically coupling it to the nucleus. This provides a direct mechanical link from focal adhesion to the nucleus, leading to nuclear flattening, stretching of nuclear pores and thereby increasing YAP nuclear import [50]. Therefore, in stiff, osteogenic environments MSCs shuttle YAP to the nucleus to activate mechanosensitive signalling pathways.

As substrate stiffness correlates to the expression and localization of cadherins and integrins, and therefore cell–cell and cell–matrix adhesions, this mechanosensitive mechanism can be exploited to modulate biochemical signalling [97–99]. One study highlighted this by using materials modified to present adhesive peptide motifs to modulate fate commitment in MSCs (figure 3). Co-presentation of the N-cadherin adhesive sequence HAVDI, mimicking cell–cell interactions, and the FN RGD sequence, mimicking cell–ECM interactions, were integrated into hydrogels of tuned matrix stiffness. Upon co-presentation on stiff 2D gels, YAP/TAZ remains cytosolic, thereby preventing osteogenic lineage commitment. However, when the HAVDI motif is removed and MSCs are thus exposed to an environment with more ECM-like interactions, YAP/TAZ translocates and MSCs commit to osteogenesis [66]. Interestingly, however, things differ in three dimensions, when the HAV motif (the conserved part of the HAVDI sequence) is presented to MSCs encapsulated within hyaluronic acid (HA) gels, cells perceive this more cell–cell-like interface and commit to chondrogenesis—chondrocytes typically live in pairs and hence cell–cell interactions are crucial [64].

Mechanical memory may have importance and implications for cell–material interactions, but also for clinical use. MicroRNAs (miRNAs) are short, single-stranded, non-coding RNAs. They bind one or more messenger RNA (mRNA) and therefore regulate protein expression; typically, miRNAs attenuate the ability of mRNAs to be translated to protein by binding the mRNA sequence [100]. MiRNA21, a known regulator of fibrosis, is found to be elevated on stiff substrates (such as tissue culture plastic) compared to softer substrates (such as hydrogels). Elevated levels of miRNA21 repress mRNAs coding for proteins involved in anti-fibrotic actions [101]. MSCs with elevated miRNA21 levels cultured on stiff substrates (approx. 100 kPa), retain mechanical memory for several passages once moved to soft hydrogels (15 kPa). Fibrotic scarring is a major clinical problem, therefore MSC mechanical memory has clinical implications when considering use for transplantation. It should be considered during pre-culture of MSCs, stiff substrates should be avoided [101]. MiRNA21 also targets several proteins involved in osteogenesis, such as SMA Small, MAD mothers against decapentaplegic 7, SMAD7, and SRY sex determining region Y-box2, SOX2. When MSCs with reduced miRNA21 expression are pre-cultured on soft substrates, they show more osteogenic potential that those exposed to stiff substrates, this suggests that lineage potential may be reduced by the onset of a fibrotic programme [101].

Recently, a more counterintuitive mechanism of stiffness related integrin–ligand interactions has emerged. The ‘molecular clutch model’ has long been proposed to explain actin cytoskeleton and cell migration dynamics and has now been employed to explain cell mechanotransduction [102]. Using gels with controllable rigidity and ECM ligand (RGD) spacing, it was shown that at low stiffness, close to that of some of the softer body tissues (greater than 5 kPa), when spacing between ligands was increased, focal adhesion growth also increased. Remarkably, at higher stiffness ranges typical of many body tissues, increased RGD spacing lead to focal adhesion collapse. At 30 kPa the rigidity threshold was found at around 100 nm RGD spacing, and at 150 kPa focal adhesion collapse was found at 50 nm spacing. Above this stiffness range, i.e. that of tissue culture plastic, conventional rules apply—increased ligand density is required for adhesion formation. However, for these more physiological stiffnesses, the molecular clutch can be employed, that is, ligand spacing is not via direct sensing, but instead individual integrin–ECM ligands (or ligand–cell adhesion molecule) act as the ‘molecular clutches’ and as force load increases more clutches are recruited, up to a threshold value. The recruitment of more clutches redistributes the force load among them, thus reducing the total force each individual clutch is exposed to. When the threshold recruitment is reached at high stiffness and increased spacing, no further distribution can occur and the adhesion collapses [103].

Other key stiffness-related mechanisms are also becoming elucidated with the use of biomaterial strategies. In 2013 Swift *et al.* linked the mechanical tension exerted on a cell through tissue specificity and ECM stiffness to changes in transcriptional programmes. They proposed a model supporting a physical link between the nucleus and mechanical properties of the extracellular environment, whereby tension from the ECM lead to biophysical changes in the cell cytoskeleton on the nucleus [104]. Cells discern, translate and transmit mechanical cues biochemically through mechanosensitive receptor-mediated signalling pathways [105], and alternatively cell–cell and cell–ECM interactions are interwoven with adhesion and filament networks to transmit forces. Further, cytoskeletal rearrangement due to substrate stiffness can distort the nuclear envelope and therefore alter chromosomal positioning within the nucleus, altering the spatial access of transcriptional regulators to distinct chromatin sites [51]. Swift *et al*. showed that environmental stiffness can change the transcription of the gene *LMNA*, and the stability of its protein lamin-A. In response to increased cellular tension, turnover of lamin-A is reduced leading to physical stiffening of the nucleus and stabilization of the nuclear lamina and chromatin, and thus accumulation of YAP [104].

To describe how the cytoskeleton can transmit such forces, elegant, tension based, cell-tensegrity models have been proposed by Inger [106–108]. His group have illustrated direct mechanical connection from integrins to the nucleoplasm [109] and, indeed, mechanical interconnection between the chromosomes [110]. Further evidence of direct cytoskeletal–nucleoskeletal coupling can be evidenced through linkers of cytoskeleton and nucleoskeleton (LINCs [111]) and also matrix attachment regions (MARs) between the nuclear lamins and the telomeric regions of the chromosomes [112]. Using magnetic integrin stimulation (magnetic nanoparticles coupled to integrin receptors with applied magnetic fields—magnetic twisting cytometry), Inger and colleagues have been able to show that integrin twisting can distort the nucleus [113,114].

The last decade has focused heavily on tuning and defining matrix stiffness of biomaterial niches; it has been a key facet in controlling cellular fate through traction forces. However, what is often neglected is that tissues and organs of the body are often not purely elastic. Non-degradable hydrogel systems capture some aspects of the physiological ECM environment, however reconstituted ECMs such as collagen and fibrin, and tissues such as adipose tissues, brain, liver, fracture haematoma, and the soft callus of regenerating bone, are all temporally viscoelastic [115]. The microenvironment can either store (elasticity) or dissipate (viscosity) cellular forces, thus impacting interacting cells. As both investigative and fabrication techniques progress exploration into three-dimensional matrices has pushed the field to more biomimetic systems, with strain exertion differing by almost an order of magnitude between two- and three-dimensional cultures (3–4% in two dimensions, 20–30% in three dimensions) [115–117], this should become a key caveat for ‘humanized’ *in vitro* modelling systems.

Because of this, there is now focus on the effects of three-dimensional matrices on MSC differentiation that go beyond bulk material mechanical properties. MSCs encapsulated in alginate of varied stiffness (2.5–110 kPa), presenting RGD motifs at various clustering densities, highlighted that stiff substrates that hindered cell spreading still enabled osteogenic commitment. Hence, rather than cell morphology it is the cells ability to recognize and cluster adhesion ligands, thus generating traction forces, that drives cell fate [118]. This supports the premise, in three-dimensions, that cells ‘sense’ differing stiffness as differing adhesion-ligand presentations.

Another key example combines both three-dimensional culture with matrix degradability; a tuneable HA based system that either permits or prevents cell-mediated degradation was used to show cell fate is morphology-independent when the matrix is degradable, but is instead directed by cell-mediated traction forces. Local degradability allowed cells entrapped in gels to reach otherwise unavailable adhesive ligands, rearranging their cytoskeletal structure to generate traction forces that potentially lead to osteogenic commitment, irrespective of morphology or bulk matrix stiffness [119]. These two studies suggest that cell-mediated traction forces are influenced by the cells' ability to locally deform rigid substrates but show that bulk matrix rigidity directly [118] or indirectly [119] regulates cell fate.

Fibre architecture has also been highlighted as a mechanism for transducing matrix stiffness, where lower fibre stiffness allow cells to recruit nearby fibres and correlates with enhanced cell spreading and proliferation [120]. Dynamic properties such as matrix degradation and fibre recruitment establish the fourth dimension into three-dimensional matrices [121]. The ECM and niche are naturally dynamic microenvironments, and hence the introduction of time more accurately captures *in vivo*-like mechanical behaviour, and is also important when considering MSC mechanical memory [95]. The importance of mechanical dynamics is further highlighted by a recent study, where controlled stress-relaxation of substrates determined MSC fate changes; the rate of the stress relaxation determined the degree of mechanical matrix remodelling. In rapidly relaxing hydrogels, MSCs undergo osteogenesis due to the strain the cells exert on the hydrogel introducing an initial level of intracellular stress that eases with matrix reorganization. This facilitates integrin clustering which in turn generates tension allowing the cell to ‘sense’ the stiffness of the material; leading to morphology changes and bone matrix formation [115]. The incorporation of these dynamic features into three-dimensional matrices will be critical in understanding and harnessing the cellular response to mechanical properties.

### 2.3. Chemistry

Chemistry-based strategies to engineering substrates have also been employed, changes in cell adhesion to substrates can be controlled by manipulation of properties of materials such as surface wettability [122,123], and through control of surface properties that influence protein adsorption and thus are able to enhance and control cell adhesive properties [37,124,125]. It has been shown that different chemical groups can influence MSC fate, for example, poly(ethylene glycol) (PEG) hydrogels functionalized with small molecule chemical groups are able to direct adipogenesis with hydrophobic *t-*butyl groups, and osteogenesis with charged phosphate groups [126]. However, organic chemistry is beginning to offer new strategies for biomaterial synthesis and control, for example developments in click-chemistry [127–129] and GF tethering techniques [130,131], it offers a diverse toolbox for precise functionalisation of substrates.

Commonly used PEG hydrogels can be modified to contain photodegradable cross linkers, allowing the user post-gelation control over substrate properties such as gel conformation and biochemical composition with MSCs *in situ*. Photodegradation of the hydrogel network changes the physical three-dimensional microenvironment of the encapsulated cells and can lead to migration or lineage commitment [132]. Incorporation of light-controlled binding moieties, such as RGD, can also lead to dynamic control over cell fate; hydrogels were functionalized with pendular RGD motifs that are dynamically cleaved by a light stimuli, decreasing availability for adhesion formation and leading MSCs to commit to chondrogenesis [132]. Additional studies apply other click-chemistry strategies to design protease-degradable PEG hydrogels, these incorporate locally sequestered GFs only released upon cell infiltration [133].

Similarly, enzymatic activation was exploited in a 2D PEG based system. Here RGD groups were attached to a low-adhesion PEG or Fmoc (fluorenylmethyloxycarbonyl), the ‘blocking group’ via an elastase cleavable linker, with the blocking group preventing easy access to the RGD moieties for cell integrins. With the blocking group in place, adhesion and intracellular tension were lowered sufficiently to support MSC self-renewal. However, upon elastase cleavage of the dialanine (AA) linker, cells could access the RGD more easily, form larger adhesions supporting greater intracellular tension, driving osteogenic differentiation [134].

Other chemical approaches aim to create ECM-mimetic materials, the FN RGD adhesion domain has been incorporated into many materials, however, when it is presented with its synergy domain PHSRN it enhances the affinity of integrin binding over 40-fold [135]. This FN fragment, FNIII7-10, can be engineered to retain the native spacing between the synergistic sites, which increases specifically the binding of integrin α5β1. Surfaces engineered that contained RGD-only, or failed to control site distances, showed a decrease in cell adhesion [136]. This control over integrin binding using FN fragments was found to promote osteoblast differentiation *in vitro*, and improve osteointegration of titanium implants *in vivo* [136–138]. Such adhesive group grafting has similarly been used to functionalize titanium. Here, the RGD motif was presented with PHSRN to induce efficient osteogenesis of MSCs [75]. Titanium is the gold standard material for hip replacements and many other orthopaedic materials, and hence this study provides an example of how materials already employed in a clinical setting could be simply functionalized to create cell instructive materials that may increase implant efficacy.

**Figure 4.**
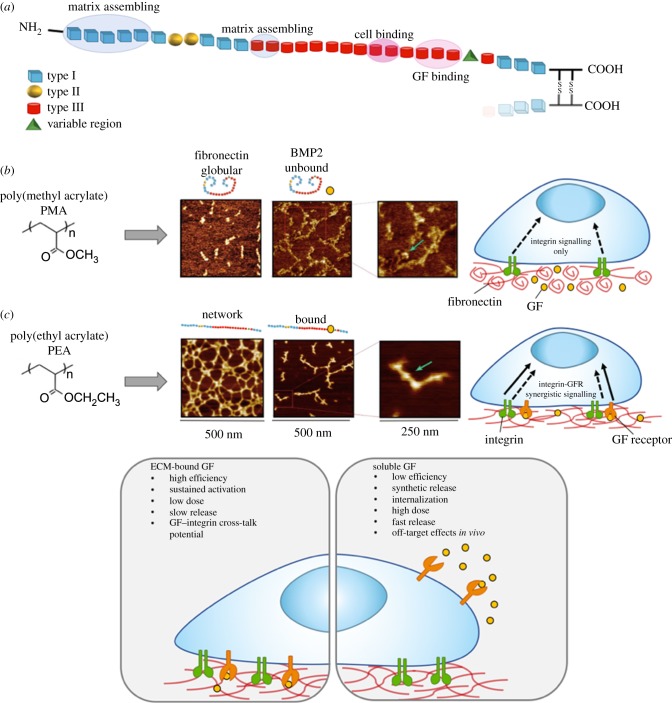
Fibronectin (FN) nanonetwork formation and growth factor (GF) presentation. (*a*) FN has three types of domains involved in FN–FN interactions (I_1–5_), GF sequestration (III_12–14_) and integrin binding (III_9–10_). (*b*) Atomic force microscopy (AFM) images after FN adsorption shows spontaneous formation of FN nanonetworks on PEA, but not PMA which results in globular FN aggregates. Sequential adsorption of growth factor (here BMP-2, at 25 ng ml^−1^) shows interactions of BMP-2 with FN fibrils on PEA, but not PMA, due to an open molecular conformation exposing the key GF binding domain. The proximity of the GF binding and integrin binding domain on FN molecules in networks leads to synergistic integrin and GF receptor signalling. (*c*) Conventional solubilized GF delivery (right) normally requires high dosages, making it less efficient than ECM-bound (or solid-phase presenting) systems (left) that may use several orders of magnitude lower dosages, allowing efficient, targeted delivery that also has the potential to facilitate cross-talk between integrins and growth factor receptors. (*a*) Adapted from and (*b*) taken from reference [130]. © The Authors, some rights reserved; exclusive licensee American Association for the Advancement of Science. Distributed under a Creative Commons Attribution License 4.0 (CC BY). (*c*) Adapted from reference [65]. (Online version in colour.)

In addition to its integrin binding domains, FN also contains a highly promiscuous GF binding domain in its 12th to 14th type III repeats [139] (figure 4*a*), meaning this molecule could also be exploited as an approach to deliver GFs. GFs have been widely employed in the clinic, due to their capacity to regulate cell growth, healing and stem cell differentiation, however soluble GF administration usually means application of supraphysiological doses which can lead to serious off-target side effects [140]. One study engineered a recombinant FN fragment to contain both the integrin binding and the GF affinity domain bound to a fibrin matrix, leading to potent synergistic signalling through recruitment of integrins and GF receptors to adhesion domains. By incorporating platelet-derived growth factor (PDGF) and bone morphogenic protein 2 (BMP-2), they found the system could promote both wound repair and bone growth [141]. This recombinant protein approach to exploit FN integrin-binding and GF-sequestering domains is employed as upon adsorption to synthetic materials FN typically adopts a globular conformation, concealing these binding sites. However work by our group found that upon FN adsorption, the polymer poly (ethyl acrylate) (PEA) causes spontaneous unfolding of FN leading to assembly into nanonetworks, thus exposing cell binding and GF binding domains (figure 4*b*) [142]. This approach allowed absorption of BMP-2 at approximately 300-fold lower dose than the current gold standard application for bone repair. This simple engineering approach exploits the synergistic presentation of BMP-2 on the FN material-driven networks, and was shown to drive significant bone regeneration in a critical size defect *in vivo* [130]. This system has also been used to coat titanium implants to present ultralow doses of BMP-7, with the aim to improve the bio-integration of titanium implants [143], and further to this, by tethering vascular endothelial growth factor (VEGF) improved vascularization of biomaterial scaffolds was achieved *in vivo* [144].

Immobilized GF technologies not only permit lower doses of the GF to be employed (thus being more cost effective than solubilized GF delivery) but as many of these ligands act at interfaces *in vivo*, immobilization also permits a more biomimetic recapitulation of stem cell niches [131,145] (figure 4*c*). Controlled tethering of such ligands to synthetic materials has proved difficult [146], as non-specific cross-linking may compromise the molecules bioactivity and further issues in controlling the amount, stability and cellular accessibility to the ligands. A solution to this was presented by Fan *et al.*, were a poly(ethylene oxide) (PEO) based co-polymer was used to covalently tether epidermal growth factor (EGF) to the substrate, a GF associated with tissue repair. Specific tethering at the N-terminus retained EGF bioactivity but restricted it to the surface. EGF tethering led to signalling through its receptor EGFR, in a manner akin to physiological-like matrix-embedded EGF, where EGFR is stimulated but not internalized [147]. Another striking example of protein tethering is immobilized leukaemia inhibitory factor (LIF), an essential protein for ESC self-renewal, to maleic anhydride copolymer thin-film coatings. Here, they showed retention of pluripotency for at least two weeks in absence of soluble LIF in culture media (i.e. standard ESC feeder-free culture conditions) [131].

More recently, block copolymer micellar nanolithography was used to coat substrates with evenly spaced and tuneable arrays of gold nanoparticles functionalized to carry BMP-2 molecules. This system allows precise control over the nanoscale distribution of the BMP-2, with one BMP-2 molecule/nanoparticle, both the local amount and the distance between BMP-2 anchor points can be varied. Here it was found that immobilized BMP-2 increased intracellular Smad-signalling compared to if the GF was administered soluble in the media, even at concentrations as low as 0.2 ng cm^−2^ Smad-dependent signalling was observed [148]. This system permits sustained local delivery of BMP-2 or other GFs, and allows the precise study of the effects of GF density and spacing on intracellular signalling.

## 3. Artificial stem cell niches for self-renewal

Using biomaterial interfaces, mechanisms for MSC differentiation are beginning to be well understood. However, understanding MSC self-renewal *in vitro* is of growing importance. MSCs are increasingly used in tissue engineering strategies, but are also being studied for their tumour-homing abilities for drug delivery [5] and as anti-inflammatories to modulate graft-versus-host disease [8,9,14]. To support this, MSCs need to be isolated and expanded *in vitro*, this presents a challenge due to loss of self-renewal capacity on normal tissue culture plastic.

*In vivo* this homeostasis between self-renewal and differentiation, quiescence and proliferation, is tightly controlled by many factors from the niche (figure 1). Through modulation of biomaterial properties discussed above, *in vitro* stem cell self-renewal is being investigated. As noted earlier, nanotopographies have been employed with well-defined patterning, an ordered square pattern leads to retained multipotency of MSC markers over long culture periods [44]. Muscle stem cell regenerative capacity has been maintained in culture microenvironments that mimic the native stiffness of muscle (12 kPa) [149], and simple chemical modification of glass substrates have been used to present functional groups that maintain MSC phenotype [150]. It is notable that control of MSC self-renewal by stiffness remains elusive. However, it has been demonstrated that environments with homogeneous stiffness do not support cell growth as well as heterogeneous environments. Using photodegradable hydrogels, it has been shown that disorganized patterns of matrix mechanics lead to cytoskeletal disruption, decreased cell spreading and prolonged expression of MSC-related marker proteins [151]. Further, nanoparticle based approaches have been used to maintain MSC phenotype. Using super paramagnetic nanoparticles, MSCs were magnetically levitated into spheroids within collagen type I gels. In this three-dimensional niche, they remained quiescent and expressed niche/MSC markers such as nestin and stro-1. Further, using a simple wound healing model that involved placing the spheroid-niches above monolayers of different phenotypes (fibroblast, osteoblast, chondrocyte) that were subsequently scratched, the cells could respond to the regenerative demand with migration, differentiation and engraftment into the required phenotype [152]. It is important to note that if intact, non-scratched, monolayers were used, the MSCs in the niches remained quiescent. This is a key observation of a regeneration responsive, but otherwise quiescent three-dimensional *in vitro* niche.

Understanding the mechanisms behind adult stem cell, and specifically MSC, self-renewal has been somewhat limited. For MSCs, self-renewal requires an intermediate adhesion state that suppresses differentiation and allows for long-term growth *in vitro*. As highlighted by many biomaterial strategies, osteogenesis of MSCs requires large adhesions that support high intracellular tension [43,45], with adipogenesis opposing this, occurring when adhesion is weak and tension low [39,40]; conditions favouring MSC self-renewal sit mid-way between these two fates. However, as these conditions also favour fibroblast formation, it has been hard to achieve this in culture [32,65].

MSC self-renewal mechanisms are emerging as rather different than the better known embryonic self-renewal mechanisms using NANOG, SOX2 and OCT4 [153], to dissect MSC self-renewal biomaterial strategies are being developed, for example the highly-ordered square (SQ) nanotopography [43,44,154]. Mitogen activated protein kinases (MAPKs) have emerged as the ‘switch’ that controls MSC growth and differentiation [155], extracellular signal-related kinase (ERK1/2) is a known key regulator of proliferation and growth, and along with other MAPKs is also known to act in cell cycle regulation. ERK1/2 is also implicated in phosphorylation of lineage defining transcription factors, for example, for osteogenesis [155]. It has been proposed in HSCs and pluripotent cells that the transition from early to late G1 phase is crucial for self-renewal [156–160]. Using nanotopographies, mitogenic factor cyclin dependent kinase 6 (CDK6), which is involved in G1-S transition, was found to be upregulated in proliferating MSCs [33], and has also been shown to inhibit bone morphogenetic protein (BMP)-induced osteogenesis [161], this suggests a role in both growth progression and differentiation restriction. While MSCs undergoing material-controlled self-renewal have normal growth and proliferation rates, the MSCs spend longer in G0/G1 and less time in G2 [162]. The increase in G1 phase in cells is accompanied by the repression of cyclin D1, which forms a complex with cell division cycle 2 protein (cdc2) to drive cell cycle progression [163]. Similar cell cycle control has been reported in neural stem cells [160], while pluripotent stem cells have shorter G1 phases [157–159]. It is notable that when undergoing material-controlled osteogenesis, the expression of E2F transcription factor 1 (E2F-1), which promotes G1 to S phase transition, is decreased [164], and phosphorylated retinoblastoma protein (pRB), which blocks the entry from the G1 to the S phase and which is associated with the activation of RUNX2 [165], is upregulated. This suggests that MSCs regulate cell cycle to both slow growth (osteoblasts are slow growing cells while MSCs are fast growing cells) and prime osteogenic sensitivity.

As *in vivo* stem cell niches are complex, combinatorial bioengineering techniques are moving towards allowing deconstruction and reconstruction of these multifaceted systems. To shed light on mechanisms used by stem cells in their niches, one approach is to simplify the system. To do this, microarray platforms have been developed that allow screening of the effects of unique combinations of multiple microenvironmental signals on stem cell fate. Using robotic spotting technologies mixtures of protein cues, such as ECM components, niche interaction ligands and other signalling proteins, can be presented and analysed at the single-cell level [166,167]. One example cultured human neural precursors on protein printed arrays and found cells remained in an undifferentiated state only when stimulated by two morphogens in concert, Wnt and Notch [168]. This approach has also been used to investigate ligands involved in the conversion of mammary epithelial cells to myoepithelial or luminal epithelial fates [169]. More recently, Roch *et al.* presented ligand components of the bone marrow niche and identified candidates important for HSC maintenance, presented to HSCs and using single-cell analysis, they were able to define gene expression signatures of HSCs as they differentiated into multipotent progenitors (MPPs) [170].

Similar microarray platforms have been developed to screen for self-renewal promoting materials discovery. Both human embryonic and induced pluripotent stem cells (hPSC) have the ability to self-renew indefinitely in culture, and hence hold great promise for drug discovery and regenerative medicine [171–174]. However, present culture methods for clonal expansion of these cells is suboptimal—involving mouse embryonic fibroblast feeder layers or animal derived feeder-free culture systems (such as Matrigel) which are inefficient, prone to both batch-to-batch variability and xenogenic contaminants [123,175,176]. Defined culture conditions need to be established to produce safer and more biomedically useful hPSCs. Combinatorial polymer libraries were first posed in 1997 [177] and in 2004 on-slide synthesis of polymer microarrays was achieved by Langer's group [178]. Rapid synthesis of acrylate based polymers is achieved by monomer combinatorial mixing printed onto hydrogel-coated slides, which then undergo ultraviolet-photoinitiated free-radical polymerization [122,178,179]. Such polymer microarrays were subsequently used to screen a first-generation library of 496 acrylate polymer combinations [122]. Assessment of clonal growth, expression of key pluripotency marker OCT4, and characterization of surface properties (such as wettability, elastic modulus and surface roughness) were quantified using high-throughput methodologies. Through mapping stem cell behaviour to material properties, surface wettability was found to be the strongest modulator of colony forming frequency, and engagement of integrins α_V_β3 and α_V_β5 with adsorbed vitronectin promoted hPSC self-renewal for long-term culture periods [122]. Scalability of hit materials was not demonstrated in this study [122], however, it is envisioned how such synthetic, readily synthesized monomers could be scaled up to create cell cultureware, removing some of the current hurdles for biomedical translation of these cells.

## 4. Future and conclusion

Many of the approaches discussed above are reductionist in nature and have been key to elucidating and understanding key mechanisms for how cells sense and respond to their complex microenvironment, however, while these approaches are easily scalable and robust, they lack biological functionality. Recent material strategies are now beginning to re-build the complexity of the niche *in vitro* to create more tissue-like systems, as there is a strong need for more humanized, NATs (bioengineered, cellular, scaffolds, on-chip systems) for drug discovery, disease modelling and regenerative medicine.

For example, one study designed a synthetic hydrogel (PEG based) system to define the ECM parameters that govern ISC expansion and organoid formation. Organoid formation is through self-organization of stem cells into structures that resemble their native multicellular architecture and many of their functional features. Here, by designing a matrix that was mechanically dynamic, it allowed for optimal ISC expansion (stiff substrate, FN-based adhesion), and subsequently for differentiation and intestinal organoid formation (soft-matrix, laminin-based adhesion). This dynamic substrate design could thus support the need for the changing mechanical environments throughout the native cellular evolution during organ development [180]. Another key example of intestinal organoid formation was recently developed also using a modular PEG hydrogel system, here adhesive peptides are bound to one fraction of the four-armed PEG molecule to mimic basement membrane interactions, the unconjugated arms are then cross-linked with a protease-cleavable peptide to form the hydrogel. Both the adhesive sequences and the cross-linking peptides can be exchanged, and by varying the PEG concentration, matrix stiffness is also tuneable. hPSC were seeded into gels to form intestinal organoids, and upon implantation into mechanically injured colons of immunocompromised mice could enhance healing of the defects [181]. These fully synthetic systems offer well-defined alternatives to animal-derived platforms (such as Matrigel), expanding applicability as complex models for both potential therapeutic applications, and clinical and industrial research.

Further to organoids, organ-on-a-chip (OOC) platforms seek to recapitulate the multifaceted tissue-specific cellular microenvironment in three-dimensional settings for a given organ system. OOC aims not to build a whole living organ, but to establish a minimally functional unit that is representative of aspects of human physiology in a controlled system [182]. Several limitations posed by organoid systems which influence organ development and function during development and disease can be addressed with these microfluidic OOC systems—for example parameters such as physical forces including fluid shear stress [183,184], mechanical compression [185,186] and electrical stimulation [187]. OOC allows for recreation of tissue interfaces (e.g. lung alveolus–capillary [186], blood–brain barrier [188]) multicellular compilations that enable communication between multiple cell types (e.g. liver hepatocytes and fibroblasts [189,190]) and the integration of individual OOCs through microfluidic channels to mimic physiological organ subsystems (e.g. liver–pancreas [191], liver–heart [192]). By guiding or situating collections of cells that can assemble into three-dimensional structures OOCs can represent simplified yet realistic models of organ-level systems, and offer functional read-outs that suit the intended application. Systems such as these can model healthy and diseased development and behaviours, offering again more realistic models for drug discovery and toxicity testing. However, it is also envisioned that their use alongside clinical trials will help realize a precision medicine approach, where individual patients can be tested, or important differences in varied patient cohorts (such as sex, age, ethnicity, other pathology) could be considered and trials optimized for specific patient biology [182].

On the other hand, microwell formats that recapitulate cell–cell and cell–matrix interactions typical of stem cell niches will be valuable for analysis of heterogeneous populations of stem cells at the single cell level. Integration of material technologies and protein patterning will be crucial in fabrication of microwell arrays, these will allow high throughput screens to identify ECM molecules relevant to ‘niche-like’ regulation. By developing arrays that mimic cell–cell interactions through peptide presentation, single or combinations of molecules can be identified that induce effects on cell behaviours, such as self-renewal and lineage commitment [167,170]. Typical cell–cell interaction studies rely on co-culture of two or more cell types together that not only make it difficult to distinguish the key molecular effectors, but also encounter further problems such as compromised media types, multiple patient sources, and biological variation. It is envisioned high-throughput approaches could be developed to incorporate many of the biophysical and biochemical properties discussed throughout this review.

Technologies such as these will prove valuable for the pharmaceutical industry, as although in recent decades advances in the molecular understanding of diseases has underpinned new potential therapeutic targets there is a lack of corresponding increase in drug discovery and manufacture. This is irrespective of substantial increases in research and development investment, which led to a ‘volume research’ approach using new genomic and high-throughput technological approaches, and yet productivity has remained static [193]. This frustration led AstraZeneca to report that from 2011 to 2016, 66% of its small molecule projects failed; critically only 23% before clinical trials (i.e. cheap fails) [26]. This suggests that drug discovery has become more challenging, but also demonstrates a lack of ability to predict success at the pre-clinical testing stage. Further to this, although animal models are widely used for both drug discovery and toxicology screening, it is notable that 43% of new drug fails are not predicted by the *in vitro* and pre-clinical *in vivo* screens. It is envisioned that technologies such as many discussed above will solve these issues, by engineering validated NAT models that can more readily predict drug targeting and tissue dose exposure, and hence leads with high risk of failure can be dropped quickly—before expensive clinical trial stages. As discussed above, biomaterial engineered systems can easily introduce complex physiological-like parameters, removing problems of overly simple *in vitro* cell lines and *in vivo* models (non-human, animal based). Currently, regulators are encouraging the development of tissue models, such as organoids, to help provide ‘humanized’ data alongside traditional *in vivo* data [194]. Further, the banning of animal testing in toxicology screenings for e.g. cosmetics in the UK, has driven the use of NAT models for toxicological assessment. Several bioengineered approaches have emerged for skin/eye irritation and phototoxicity, for example EPISKIN™, SkinEthic RTE and EpiDerm™ [195–197].

NAT development will also play a key role in the advancement of the use of stem cells for clinical applications in regenerative medicine. Current culture systems rely on non-human (xeno) serums in culture mediums or feeder layers to support SC survival and expansion. However, these xeno systems can induce as immune response upon clinical transplantation, for example, FBS is a potential source of pathogens, such as endogenous retroviruses and xenoepitopes, and batches of the feeder layer Matrigel have been found to be contaminated with lactate-dehydrogenase-elevating virus (LDV). Further to this, xeno-derived components suffer greatly from batch-to-batch variability. This has driven searches for serum-free and feeder-free culture systems, although current systems contain several recombinant proteins or inhibitors whose long-term effects are yet to be understood. Suggesting further, the need for more humanized models that remove contaminating animal factors, it is highly anticipated that this can be approached by the advancement in understanding of the stem cell niche with biomaterial and biofabricated systems.

In conclusion, the future for materials as biological tools to dissect stem cell functions, as the building blocks to develop NATs to accelerate drug screening and to underpin regenerative medicine is very promising.

## Data accessibility

This article has no additional data.
